# Terminal Deoxynucleotidyl Transferase (TdT) Inhibiti on of Cord Blood Derived B and T Cells Expansion

**DOI:** 10.15171/apb.2017.026

**Published:** 2017-06-30

**Authors:** Sanaz Gholami, Seyede Momeneh Mohammadi, Aliakbar Movasaghpour Akbari, Ali Abedelahi, Alireza Alihemmati, Shirin Fallahi, Hojjatollah Nozad Charoudeh

**Affiliations:** ^1^Stem Cell Research Center, Tabriz University of Medical Sciences, Tabriz, Iran.; ^2^Anatomical Sciences Department, Faculty of Medicine, Tabriz University of Medical Sciences, Tabriz, Iran.; ^3^Hematology and Oncology Research Center, Tabriz University of Medical Sciences, Tabriz, Iran.

**Keywords:** Cord blood, TdT inhibition, B and T cells, Cytokines, Genistin

## Abstract

***Purpose:*** Terminal deoxynucleotidyl transferase(TdT) is a DNA polymerase that is present in immature pre-B and pre-T cells. TdT inserts N-nucleotides to the V (D) J gene segment during rearrangements of genes, therefore, it plays a vital role in the development and variation of the immune system in vertebrates. Here we evaluated the relationship between cytokines like interleukin-2 (IL-2), interleukin-7 (IL-7), and interleukin-15 (IL-15) and TdT expression in cord blood mononuclear cells and also effect of inhibition in the expansion of B and T cells derived from cord blood.

***Methodes:*** The cord blood mononuclear cells were cultured with different combination of cytokines for 21days, which they were harvested in definite days (7, 14 and 21) and evaluated by flow cytometry.

***Results:*** Our data indicated that TdT expression increased in cord blood mononuclear cells using immune cell key cytokines without being dependent on the type of cytokines. TdT inhibition reduced both the expansion of B and T cells derived from cord blood and also declined the apoptosis and proliferation. Considered together, TdT played an important role in the control of the expansion of B and T cells derived from cord blood.

***Conclusion:*** considered together, it was observed that TdT expression was increased by cytokines and TdT inhibition not only reduced B and Tcells derived from cord blood, but it also affected the rate of apoptosis and proliferation.

## Introduction

Terminal deoxynucleotidyl transferase (TdT) is a nuclear enzyme in one unique parcel of the pol X family of DNA polymerase.^1,2^ In human, TdT activity is present in the immature fraction of thymocytes and bone marrow cells.^3,4^ TdT plays a vital role in the development and variation of the immune system in vertebrates.^5-7^ TdT contributes to the variation of antigen receptors by random addition of nucleotides to single-stranded DNA at the junctions recombination of immunoglobulin heavy-chain genes in B and T cells development.^8-11^

V(D)J recombination creates diversity of antigen receptors in immune system.^12^ TdT is responsible for inserting nucleotides to the junctions of gene segment during V (D) J recombination.^13-15^ TdT gene is present in pre-B and pre-T cells and increases in some human leukemia’s.^9,16^ For this reason, TdT is an effective biochemical marker for classifying leukemia’s.^17^

TdT has been identified in childhood and adult acute lymphoblastic leukemia (ALL) and often appear in chronic myelogeneos leukemia (CML), acute nonlymphocytic leukemia (ANLL) and acute myeloid leukemia (AML). Also TdT expression level is elevated in Non-Hodgkin’s lymphoma (NHL) patients.^16-19^

Interleukin-2 (IL-2), interleukin-7 (IL-7), and interleukin-15 (IL-15) are certain cytokines which play an important role in generation, hematopoiesis and differentiation of hematopoietic stem cells (HSCs).^20,21^ IL-2 acts in hematopoiesis and proliferation of T cells.^22-24^ IL-7 affects the B cells development and T cells differentiation.^25^ Additionally, IL-15 influences the homeostasis, development and proliferation of T cells.^26^

It seems there are clear interactions between TdT and B and T cell rearrangements and immune cell development and hemostasis. It is important to study these contexts in cord blood since umbilical cord blood contains a plentiful source of CD34 positive cells that can be used in HSC-transplantation in immunotherapy for different cancers. Therefore, it could probably be applicable in combination of TdT inhibition for leukemia treatment. However the role of TdT in the generation of cord blood derived B and T cells is unclear.

The aim of this study is to evaluate TdT expression in cord blood mononuclear cells using different key immune cell cytokines like IL-2, IL7, and IL15. Moreover, it is to indicate the influence of TdT inhibition by genistin on the generation of B and T cells.

## Materials and Methods

### Sample collection 

Cord blood samples were collected in 50 mL falcon tubes from full-term normal deliveries by using sterile techniques in Tabriz Alzahra hospital (Tabriz city, East Azerbaijan). Ficoll-Hypaque (GE Healthcare, Piscatta, NJ, USA) was used for mononuclear cells (MNCs) isolation. Cord blood was diluted 1:2 with phosphate buffered saline (PBS, pH 7.4) and 10% fetal bovine serum (FBS), centrifuged at 850 g for 25 min, buffy coat layer was collected with syringes, washed twice and re-suspended in RPMI1640 (Gibco) with 10% FBS (Gibco) and collected MNCs were either used for culture or kept for freezing.

### Monoclonal antibodies and flow cytometry

Monoclonal antibodies, conjugated with different fluoro-chromes, were used to stain cell surface markers including: anti-CD3 (UCHT1; R&D, Minneapolis, MN, USA), anti-CD20 (clone 2H7; BD Biosciences), anti- KI67(clone: 20Raj1, eBioscience, CA 92121, USA), anti-caspase3 (Cat:51-68655X; BD Biosciences) and TdT (cat:51-35404X.4; BD Biosciences). Matched isotype control with appropriate fluro-chrome was used. Propidium Iodide (1.0mg PI/ml; Invitrogen) was used to exclude dead cells from analysis. Cells were incubated with related antibodies for 20 minutes. Afterwards samples being analyzed on FACSCalibur (BD Bioscience), between 30000 and 80000 events were collected and data analysis was performed using FlowJo software.

### Cell culture and cytokines 

The 5 × 10 ^5^ cord blood MNCs were plated in 96- well plates in 250 µL of RPMI1640 (Gibco) plus 20% Fetal Bovine Serum (FBS; Gibco) and containing 1% penicillin/streptomycin (Gibco) supplemented with cytokines with final concentrations(50 ng/mL for each cytokine): SCF, FLT3 ligand(FL), IL-7, IL-15 , and IL-2 (all purchased from PeproTech, NJ, United States). Cells were kept at 37 °C for 21 days, and half of the co-culture medium was replaced weekly. Cells were harvested in definite days (7, 14 and 21) and evaluated for different properties.

For inhibition of TdT expression, MNC cells harvested on the 21st day of the culture, incubated with 50 μM of genistin (Santa Cruz biotechnology) for 48 hours in incubator (37°C, 5% Co2). Afterwards, the samples were evaluated for different aims by flow cytometry.

## Results

### Effects of selected cytokines on TdT expression

**Figure 1:**
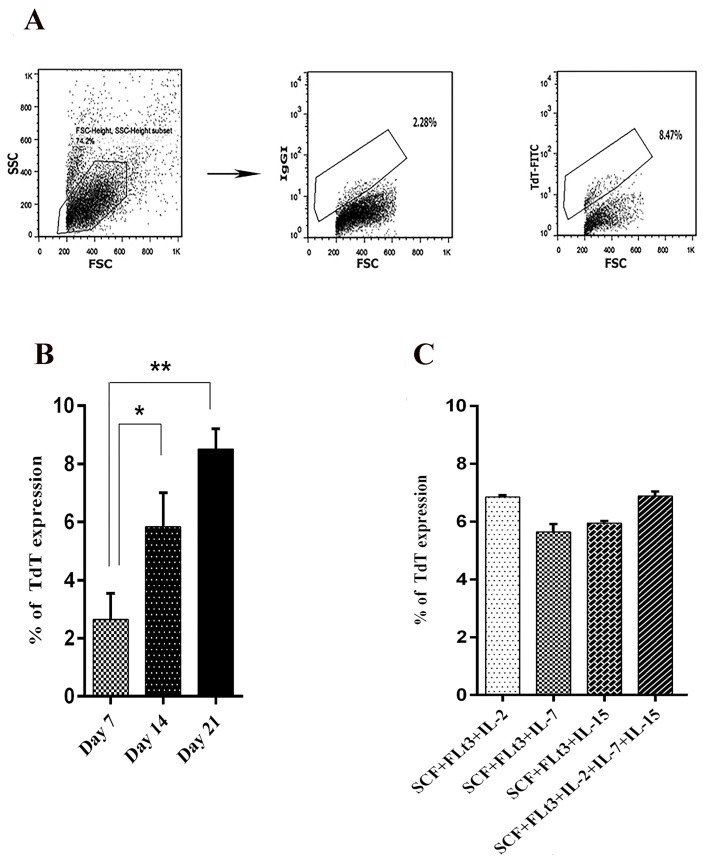
TdT expression in cord blood mononuclear cells. Representative FACS plots were used to evaluate percentage of TdT expression in indicated time points (A). TdT expression in indicated time points (culture supplemented with SCF+FLt3 (B) TdT expression using different combination of indicated cytokines in day 14(C). Values shown are mean ± SD from 3 independent experiments with 25 to 36 wells analyzed. Differences between groups are significant at (*p = 0.0379), (**p= 0.0158).

We cultured 5 × 10 ^5^cord blood mononuclear cells with different combinations of cytokines including SCF, FLt3, IL-2, IL7 and IL-15 for 21days. Harvested cells were evaluated for TdT expression (Figure 1A).

The percentage of TdT expression increased from day 7 to 21. TdT expression was around 3 and 6 in days 7 and 14 respectively, and reached to 8 on day 21 (p = 0.0379), (p = 0.0158) (Figure 1B).

However, there was no significant difference between the types of cytokines with the TdT expression. The percentage of TdT expression in the different conditions using IL2, IL7 and IL15 was around 6% on day 14 (Figure 1C).

### The impact of TdT inhibition on B and T cells

**Figure 2:**
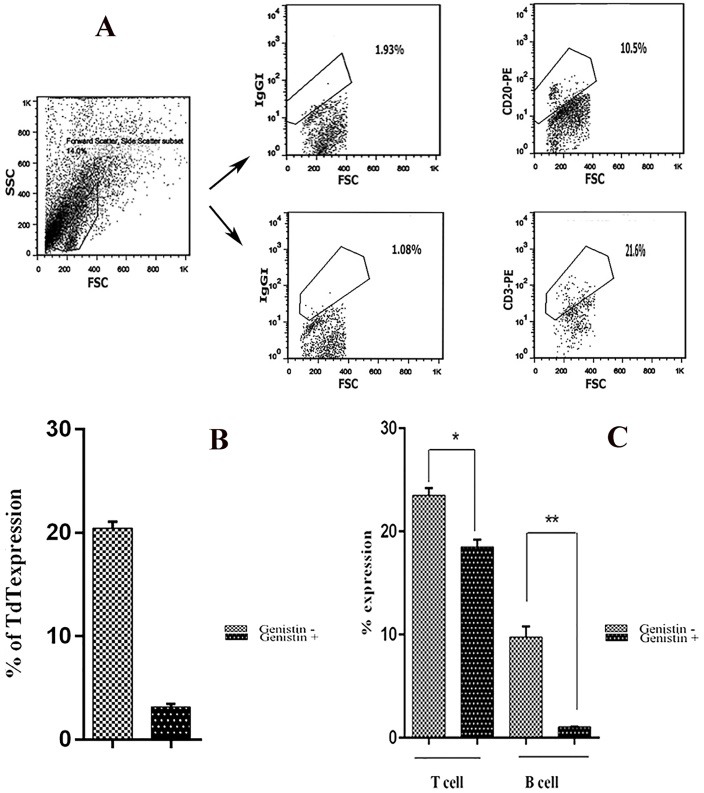
Inhibition of TdT on cord blood derived B and T cells. TdT expression following TdT inhibition using genistin (B) Values shown are mean ± SD from 5 independent treatment. Expansion of B and T cells following using genistin in day 21 supplemented with SCF+FLT3+IL-2+IL-7+IL-15 cytokines(C). Culture incubated with genistin (genistin+), culture incubate without genistin(genistin-). Values shown are mean ± SD from 8 independent experiment (*p = 0.0194), (**p = 0.0074).

As a previous study stated, genistin inhibits TdT in B and T cells development.^27^ In this study, we cultured mononuclear cells for 21 days and continued incubation for 48 hours with genistin. We showed that TdT expression reduced about 17% (Figure 2A).

Harvested cells were evaluated for percentage of T and B cells. T and B cells expression was reduced by TdT inhibition, although the reduction in B cells expression(from 10 to 1%) was significantly more than the decrease in T cells expression (p = 0.0194), (p = 0.0074) (Figure 2B).

### The impact of TdT inhibition on apoptosis and proliferation 

**Figure 3:**
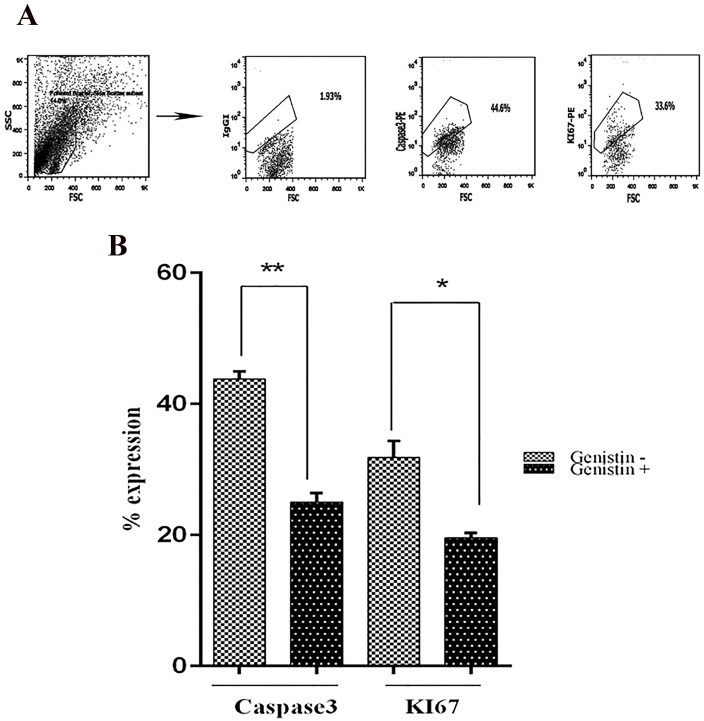
Genistin reduced apoptosis and proliferation in cord blood mononuclear cells. Representative FACS profiles for evaluation of KI67 and caspase 3 expression (A). Percentage of KI67 and caspase 3 expression in cord blood mononuclear cells following TdT inhibition using genistin (B) Culture incubated with genistin (genistin+), culture incubate without genistin(genistin-). Values shown are mean ± SD from 8 independent experiments (*p = 0.228), (**p = 0.0069).

Harvested cells were evaluated with flow cytometry at day 21 (Figure 3A). As genistin is a candidate inhibitor for leukemia, it could alter proliferation and differentiation. We evaluated caspase3 and KI67 expression for apoptosis and differentiation respectively in cord blood mononuclear cells using genistin for TdT inhibition.Percentage of caspase3 expression reduced from 44 to 26% (Figure 3B). Also KI67 expression reduced from 35 to 19% (p = 0.228), (p = 0.0069) (Figure 3B).

Inhibition of TdT by genistin significantly reduced both apoptosis and proliferation.‏

## Discussion

Terminal deoxynucleotidyl transferase (TdT) or terminal transferase as a DNA polymerase is expressed in pre B, pre T cells and in acute lymphoblastic leukemia (ALL) cells. recombination-activating genes (RAGs) and TdT are composite elements of V(D)J rearrangement.^28^ RAG-1 and RAG-2 proteins are present at breaks of double-strand at the border of recombination signal sequence (RSS, #250) and a coding segment during V(D)J rearrangements.^29^ T cell receptors (TCR) as an analogous to immunoglobulins as well as B cell receptor (BCR) are complex. The diversity of T and B cells receptors are ascribable to the junctional diversity generated during gene recombination.^30,31^ In BCR and TCR genes, TdT adds N-nucleotides to the V, D, and J exons during the gene rearrangements due to diversity and their important role in the evolution of immune cells.

In the present study, TdT expression was evaluated in cord blood mononuclear cells in presence of SCF and Flt3 as well as additional IL-2, IL-7 and IL-15. We found that TdT expression increased using different cytokines; however, it was not dependent on the type of cytokines. Inhibition of TdT by genistin reduced B and T cell expansion. As well as reducing apoptosis and proliferation.

Apoptosis regulation is important for the both development and maintenance of the immune system.^32,33^ Interleukins, including IL-2, IL-4, IL-7 and IL-15, extensively effect on lymphocytes survival during V(D)J rearrangement and later in cellular homeostasis,^34-36^ and play an important role in lymphoid cell development.^37^ It is well documented that IL-7 has important role in T cell development and B cell differentiation.^38-40^ As well as in activation of RAG gene.^41^ IL-2 can stimulate T cell proliferation.^42^ and IL15 can be useful for generation of T cells.^43,44^ Therefore probably these cytokines have key role in TCRβV-D-J rearrangements and also could influence on TdT expression.^37^

Based on above information, there are some interactions between cytokine, TdT activity and B and T cell development. Furthermore proliferation and apoptosis are the key cell activity to balance the B and T cell development and hemostasis, therefore B and T cells could be effect by any TdT alter. To sum up, TdT is a factor that interacts and influenced by cytokines, and also could effect on the rate of apoptosis and proliferation to balance B and T cell development. As shown TdT expression increases in lymphoma,^45^ maybe these findings are beneficial in the treatment of lymphoma. The inhibition of TdT could play an important role in cancer therapy, particularly in parallel with cord blood stem cell transplantation.

## Conclusion

All taken into consideration, it was found out that not only TdT expression increased by cytokines and TdT inhibition decreased B and T cells derived from cord blood, but also it altered the rate of proliferation and apoptosis.

## Acknowledgments

The authors thank the Stem Cell Research Center, Tabriz University of Medical Sciences and also appreciate our colleagues in the Anatomical Science Department (Grant code: 5/104/624,research Ethical code: TBZMED.REC.1394.558).

## Ethical Issues

Not applicable.

## Conflict of Interest

All authors declare complete responsibility of the content of the study with no conflict of interests.
